# Echocardiographic diagnosis of atrial cardiomyopathy allows outcome prediction following pulmonary vein isolation

**DOI:** 10.1007/s00392-021-01850-x

**Published:** 2021-04-29

**Authors:** Martin Eichenlaub, Bjoern Mueller-Edenborn, Jan Minners, Martin Allgeier, Heiko Lehrmann, Juergen Allgeier, Dietmar Trenk, Franz-Josef Neumann, Nikolaus Jander, Thomas Arentz, Amir Jadidi

**Affiliations:** Division of Cardiology and Angiology II, University Heart Center Freiburg-Bad Krozingen, Suedring 15, 79189 Bad Krozingen, Germany

**Keywords:** Echocardiography, Atrial strain, Atrial cardiomyopathy, Atrial fibrillation, Pulmonary vein isolation, Arrhythmia recurrence

## Abstract

**Background:**

Relevant atrial cardiomyopathy (ACM), defined as a left atrial (LA) low-voltage area ≥ 2 cm^2^ at 0.5 mV threshold on endocardial contact mapping, is associated with new-onset atrial fibrillation (AF), higher arrhythmia recurrence rates after pulmonary vein isolation (PVI), and an increased risk of stroke. The current study aimed to assess two non-invasive echocardiographic parameters, LA emptying fraction (EF) and LA longitudinal strain (LAS, during reservoir (LASr), conduit (LAScd) and contraction phase (LASct)) for the diagnosis of ACM and prediction of arrhythmia outcome after PVI.

**Methods:**

We prospectively enrolled 60 consecutive, ablation-naive patients (age 66 ± 9 years, 80% males) with persistent AF. In 30 patients (derivation cohort), LA-EF and LAS cut-off values for the presence of relevant ACM (high-density endocardial contact mapping in sinus rhythm prior to PVI at 3000 ± 1249 sites) were established in sinus rhythm and tested in a validation cohort (*n* = 30). Arrhythmia recurrence within 12 months was documented using 72-h Holter electrocardiograms.

**Results:**

An LA-EF of < 34% predicted ACM with an area under the curve (AUC) of 0.846 (sensitivity 69.2%, specificity 76.5%) similar to a LASr < 23.5% (AUC 0.878, sensitivity 92.3%, specificity 82.4%). In the validation cohort, these cut-offs established the correct diagnosis of ACM in 76% of patients (positive predictive values 87%/93% and negative predictive values 73%/75%, respectively). Arrhythmia recurrence in the entire cohort was significantly more frequent in patients with LA-EF < 34% and LASr < 23.5% (56% vs. 29% and 55% vs. 26%, both p < 0.05).

**Conclusion:**

The echocardiographic parameters LA-EF and LAS allow accurate, non-invasive diagnosis of ACM and prediction of arrhythmia recurrence after PVI.

**Graphic abstract:**

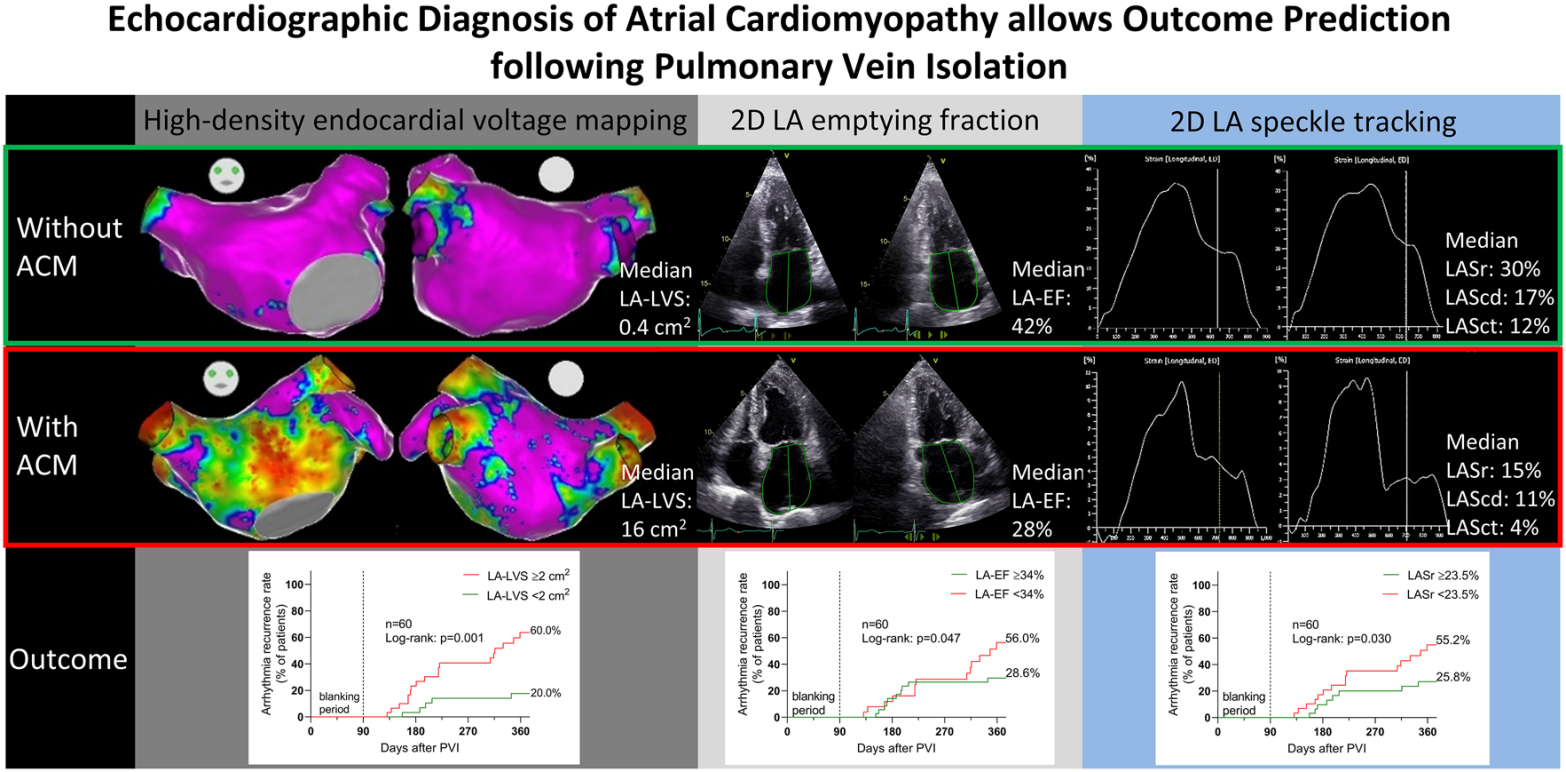

**Supplementary Information:**

The online version contains supplementary material available at 10.1007/s00392-021-01850-x.

## Introduction

Atrial fibrillation (AF) is the most common supraventricular arrhythmia worldwide with increasing incidence [1, 2]. Persistent forms of AF are associated with atrial cardiomyopathy (ACM) [3–5]. ACM, in turn, was demonstrated to be related to higher arrhythmia recurrence rates after pulmonary vein isolation (PVI) and an increased risk of stroke [6–10]. The current ESC guidelines on AF emphasize the importance of ACM diagnosis for therapeutic management and guidance of PVI [2]. Invasive endocardial contact mapping of the left atrium has been established for detection and quantification of ACM [7, 8, 11]. As a non-invasive diagnostic tool, late gadolinium-enhanced magnetic resonance imaging (MRI) was introduced over the last years [6, 9, 10]. However, the widespread use of both is limited due to clinical and practical restrictions.

Recently, echocardiographic markers of left atrial (LA) function were reported to be associated with new-onset AF and arrhythmia recurrence following PVI [12–17] and may be useful for the diagnosis of ACM [18–22].

The aim of the current study was to validate transthoracic echocardiography (TTE) in relation to high-density endocardial contact mapping in a homogenous population of ablation-naive patients with persistent AF and determine cut-off values for ACM diagnosis to identify patients at risk for arrhythmia recurrence following PVI.

## Methods

A total of 60 consecutive patients undergoing their first PVI for persistent AF were included in this prospective study. After completion of the first recruitment phase involving 30 patients, the derivation cohort, an interim analysis was performed. Subsequently, the validation cohort (*n* = 30) was enrolled. All patients underwent electrical cardioversion 4–6 weeks prior to PVI. In case of AF recurrence, patients were again electrically cardioverted in the early morning at admission. Subsequently, a TTE (GE ultrasound system E95, M5Sc probe, GE Healthcare, Solingen, Germany) was performed in sinus rhythm in the late afternoon one day prior to PVI in all patients. The following day, high-density voltage maps were acquired in sinus rhythm using an endocardial contact mapping system (CARTO-3, Biosense Webster, Irvine, CA, USA) prior to PVI. All patients underwent follow-up visits 6 and 12 months after PVI to assess arrhythmia recurrence. The study was approved by the institutional ethics committee and all patients provided written informed consent prior to enrollment.

### Transthoracic echocardiography

Standardized TTE was performed in all patients during sinus rhythm in accordance with current guidelines [23]. LA diameter was measured in parasternal long-axis at end-systole. LA volume index was calculated from four- and two-chamber views using Simpson’s biplane method indexed for body surface area. Left ventricular cavity dilatation was assessed using left ventricular end-diastolic dimension (LVEDD) measured in M-mode echocardiograms derived from 2D images in the parasternal long axis. Simpson´s method was used to determine left ventricular ejection fraction (LV-EF). Left ventricular diastolic function was assessed as described previously [24]. Biplane left atrial emptying fraction (LA-EF) was measured as LA maximum volume minus LA minimum volume divided by LA maximum volume [25]. Functional mitral regurgitation was defined as ratio of regurgitation jet area to LA area ≥ 0.1 in apical four-chamber view [26]. Two-dimensional speckle tracking was analyzed in accordance with the current guidelines [27]. For calculation of the LV global longitudinal strain apical four, two-chamber, and long-axis views were used (average of the curves from the 3 apical views with a frame rate between 57 and 90 frames per second). Furthermore, LA global longitudinal strain (LAS) was automatically analyzed offline in four- and two-chamber views using TomTec software (AutoStrain, TomTec Imaging Systems, Unterschleissheim, Germany): A complete R–R cycle (end-diastole to end-diastole) was automatically selected and endocardial borders were automatically placed by the software. LAS was measured automatically in the reservoir phase (LASr, between mitral valve closure and mitral valve opening), conduit phase (LAScd, between mitral valve opening and onset of LA contraction) and contraction phase (LASct, between onset of LA contraction and mitral valve closure). LAS values were calculated as average from two- and four-chamber views.

### Endocardial contact mapping and ablation procedure

**Fig. 1:**
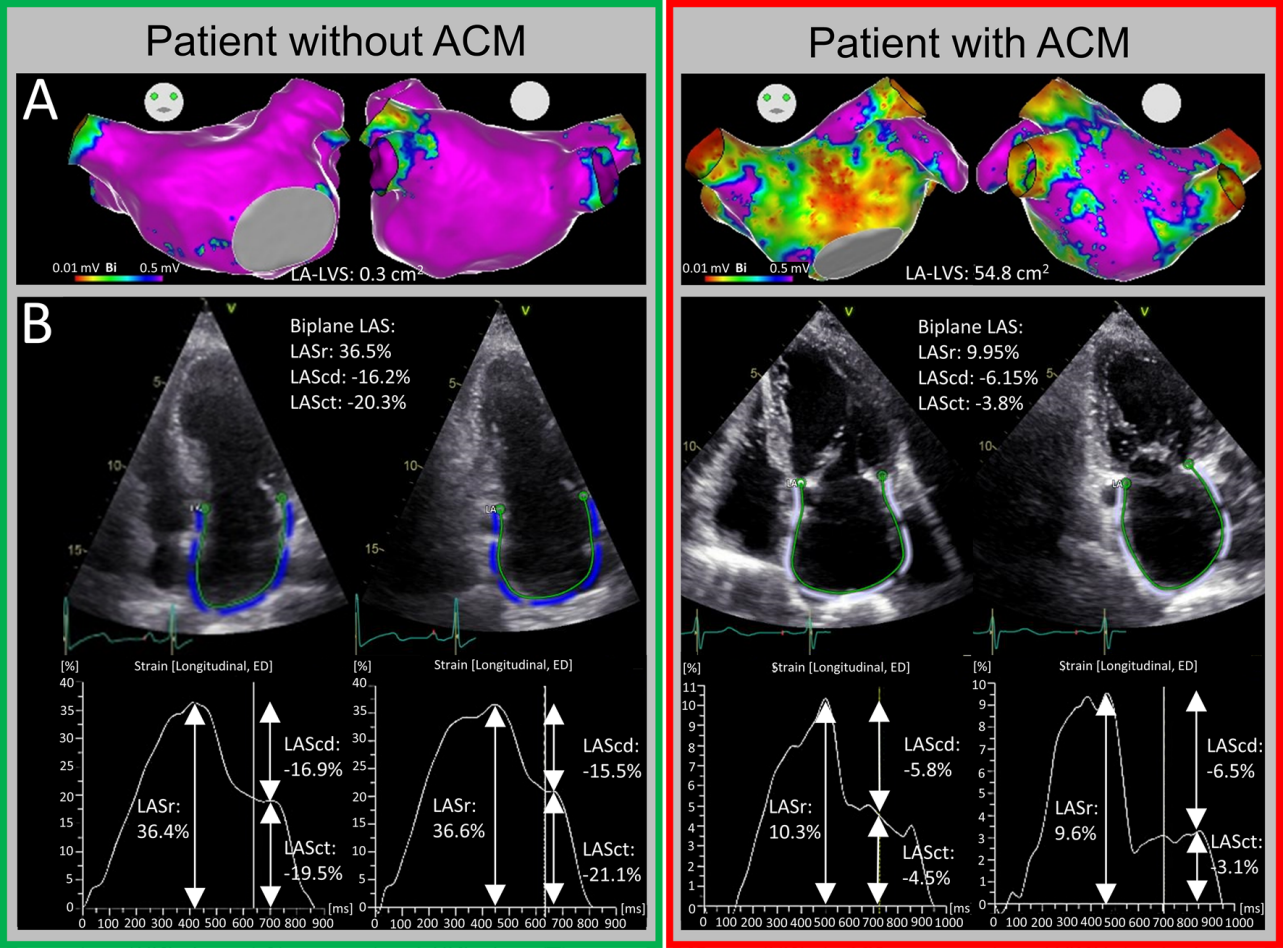
Illustration of two representative patients without and with relevant atrial cardiomyopathy. Invasive endocardial voltage map of the left atrium **(a)** and the corresponding left atrial strain (LAS) curves **(b)** are shown. Region of interest including endocardial borders was automatically determined by the speckle tracking software. On the left side, a patient without relevant atrial cardiomyopathy (ACM) with a left atrial low-voltage substrate (LA-LVS) of 0.3 cm^2^ and normal strain values (LAS in reservoir phase (LASr): 36.5%, LAS in conduit phase (LAScd): 16.2% and LAS in contraction phase (LASct): 20.3%) is shown. On the right side, a patient with relevant ACM with 54.8 cm^2^ LA-LVS and reduced strain values (LASr: 9.95%, LAScd: 6.15% and LASct: 3.8%) is depicted

High-density endocardial voltage maps (mean 3000 ± 1249 mapped sites per atrial chamber) were acquired in sinus rhythm using either a 20-polar Lasso-Nav (variable diameter: 15–25 mm) or a PentaRay-Nav catheter (electrode size: 1 mm, spacing: 2–6–2 mm, Biosense Webster, Irvine, CA, USA) in combination with the electro-anatomical contact mapping system CARTO-3 as described previously [11, 28]. ACM extent was quantified by measurement of areas with a bipolar left atrial low-voltage substrate (LA-LVS) < 0.5 mV. Relevant ACM was defined as a LA-LVS ≥ 2 cm^2^ in accordance with the prior studies [28, 29].Representative endocardial voltage maps and the respective echocardiographic measurements in patients without and with relevant ACM are illustrated in Fig. 1.

Proximal circumferential PVI was performed using an irrigated-tip contact force-enabled radiofrequency ablation catheter (Smart Touch Thermocool, tip electrode: 3.5 mm, spacing: 2–5–2 mm, Biosense Webster, Irvine, CA, USA) with an ablation index of 350–370 (at 30 W) at the posterior left atrium, 450 (at 30-35 W) within the carina and of 500 (at 35 W) at other areas and inter-lesion distances < 5 mm.

### Follow-up

All patients underwent ambulatory clinical visits including 12-lead electrocardiogram (ECG) and 72-h Holter ECG 6 and 12 months after PVI. Arrhythmia recurrence was defined as any documented episode of AF, atypical atrial flutter or atrial tachycardia lasting > 30 s after a three-month blanking period. If an arrhythmia recurrence could not be detected by 12-lead ECG and Holter ECG even though the patient was symptomatic, an event recorder was given to the patient to record a single-lead ECG during symptomatic episodes.

### Endpoints

The primary endpoints were (a) to investigate the relation of ACM and echocardiographic parameters in the derivation cohort for definition of cut-off values for ACM diagnosis and (b) to apply these cut-off values to the validation cohort.

The secondary endpoint was to assess the predictive value of the echocardiographic ACM markers regarding arrhythmia recurrence at 12 months following PVI.

### Statistical analysis

SPSS Statistics 23 (IBM, New York, USA) and Graphpad Prism 8 (Graphpad Software, San Diego, CA, USA) were used for statistical analysis. Test for normality was assessed by the Shapiro–Wilk test. Normally distributed data are given as mean ± standard deviation and skewed distributed data as median with interquartile range (IQR, 1st and 3rd quartiles). Depending on the number of groups and distribution, group comparison was performed using Student´s t-test and Mann–Whitney U test. Fisher’s exact test was used to compare categorical variables. Receiver-operating curves (ROC) were acquired to identify the optimal cut-off values for ACM diagnosis using echocardiographic parameters after inclusion of the first 30 patients and validated in the following 30 patients. Univariate linear regression analysis was conducted for all independent variables. In the multivariate model, all variables with *p* < 0.05 in the univariate models were selected for analysis. Kaplan–Meier curves were used to illustrate arrhythmia recurrence and compared using the log-rank test. Impact of clinical covariates on arrhythmia recurrence was analyzed using Cox proportional hazard regression models. A two-tailed *p* < 0.05 was considered significant.

## Results

Clinical and procedural characteristics are listed in Table 1. Patients were on average 66 ± 9 years old and 80% were males. Presence of ACM was evenly distributed with 50% of patients meeting the prespecified criteria for relevant ACM. Patients with and without ACM did not differ significantly with regard to clinical symptoms (NYHA and CCS classifications) and conventional cardiovascular risk factors such as atrial hypertension, diabetes mellitus or coronary artery disease. In both groups, only a minority of patients suffered from left ventricular dysfunction or relevant valvulopathies.

**Table 1:** Clinical and procedural characteristics

	All patients (*n* = 60)	Without relevant ACM (*n* = 30)	With relevant ACM (*n* = 30)	*p* value
Age, years	66 ± 9	62 ± 10	70 ± 6	< 0.001
Male sex, *n* (%)	48 (80)	26 (87)	22 (73)	0.33
BMI, kg/m^2^	28 ± 4	29 ± 4	26 ± 3	0.011
NYHA functional classification	2 (2–3)	2 (2–3)	2 (2–2)	0.58
CCS classification	1 (1–1)	1 (1–1)	1 (1–2)	0.65
Hypertension, *n* (%)	41 (68)	18 (60)	23 (77)	0.27
Diabetes mellitus, *n* (%)	5 (8)	2 (7)	3 (10)	1.0
Prior stroke or TIA, *n* (%)	1 (2)	0 (0)	1 (3)	1.0
Structural cardiomyopathy, *n* (%)	10 (17)	4 (13)	6 (20)	0.73
Coronary artery disease, *n* (%)	9 (15)	3 (10)	6 (20)	0.47
CHA_2_DS_2_-VASc-Score	2 (1–3)	2 (1–3)	3 (2–4)	0.004
Prior antiarrhythmic therapy, *n* (%)	49 (82)	24 (80)	25 (83)	1.0
Antiarrhythmic therapy on admission day, *n* (%)	39 (65)	20 (67)	19 (63)	1.0
Amiodarone, *n* (%) Flecainide, *n* (%) Sotalol, *n* (%) Dronedarone, *n* (%) Propafenone, *n* (%)	24 (40) 6 (10) 6 (10) 2 (3) 1 (2)	10 (33) 5 (17) 3 (10) 1 (3) 1 (3)	14 (47) 1 (3) 3 (10) 1 (3) 0 (0)	0.43 0.20 1.0 1.0 1.0
Electrical cardioversion on admission day, *n* (%)	14 (23)	4 (13)	10 (33)	0.13
LA diameter, mm	46 ± 6	44 ± 5	47 ± 6	0.019
LA volume index, mL/m^2^	49 ± 14	45 ± 12	53 ± 15	0.031
LA-EF, %	37 (26–42)	42 (36–44)	28 (17–37)	< 0.001
LASr				
4C 2C Averaged	24 (16–33) 22 (15–32) 24 (15–30)	31 (24–36) 30 (24–36) 30 (27–36)	17 (11–22) 16 (12–22) 15 (12–22)	< 0.001 < 0.001 < 0.001
LAScd				
4C 2C Averaged	15 (10–18) 14 (9–18) 14 (10–18)	17 (13–24) 17 (14–20) 17 (14–21)	12 (7–18) 10 (7–15) 11 (7–15)	0.001 < 0.001 < 0.001
LASct				
4C 2C Averaged	7 (4–13) 8 (3–14) 8 (4–13)	11 (7–17) 12 (7–19) 12 (7–18)	4 (2–7) 5 (2–9) 4 (2–8)	< 0.001 < 0.001 < 0.001
LV-EF, %	57 ± 8	58 ± 8	56 ± 8	0.552
LV dysfunction with LV-EF < 50%, *n* (%)	12 (20)	6 (20)	6 (20)	1.0
LVEDD, mm	54 ± 5	54 ± 5	53 ± 6	0.593
LV cavity dilatation, *n* (%)	12 (20)	3 (10)	9 (30)	0.10
LV strain, %	18 ± 3	19 ± 4	17 ± 3	0.097
PAP, mmHg^a^	33 (27–38)	29 (25–34)	34 (29–42)	0.028
E/A	1.48 (1.03–2.25)	1.18 (0.85–1.54)	2.16 (1.40–2.88)	0.001
DT, s	0.205 (0.180–0.240)	0.210 (0.188–0.249)	0.200 (0.170–0.228)	0.199
Septal *e*’, cm/s	7.00 (5.00–8.25)	7.00 (6.00–9.93)	6.75 (5.00–8.00)	0.061
*E/e*’	10.89 (8.37–14.00)	8.95 (7.14–11.47)	12.93 (10.65–19.69)	< 0.001
Relevant (at least moderate) mitral valve regurgitation, *n* (%)	4 (7)	1 (3)	3 (10)	0.61
Functional mitral regurgitation, *n* (%)	21 (35)	4 (13)	17 (57)	0.001
LA-LVS at < 0.5 mV, cm^2^	2.5 (0.4–17.0)	0.4 (0–1.0)	16 (4.8–26.9)	< 0.001

*ACM* atrial cardiomyopathy, *BMI* body mass index, *C* chamber, *cd* conduit phase, *ct* contraction phase, *DT* deceleration time, *EDD* end-diastolic diameter, *LA* left atrial, *LA-EF* left atrial emptying fraction, *LV-EF* left ventricular ejection fraction, *LAS* left atrial strain, *LV* left ventricular, *LVS* low-voltage substrate, *PAP* pulmonary artery pressure, *r* reservoir phase, *TIA* transient ischemic attack

^a^Measurable in 42/60 patients (70%)

### Derivation cohort

**Fig. 2:**
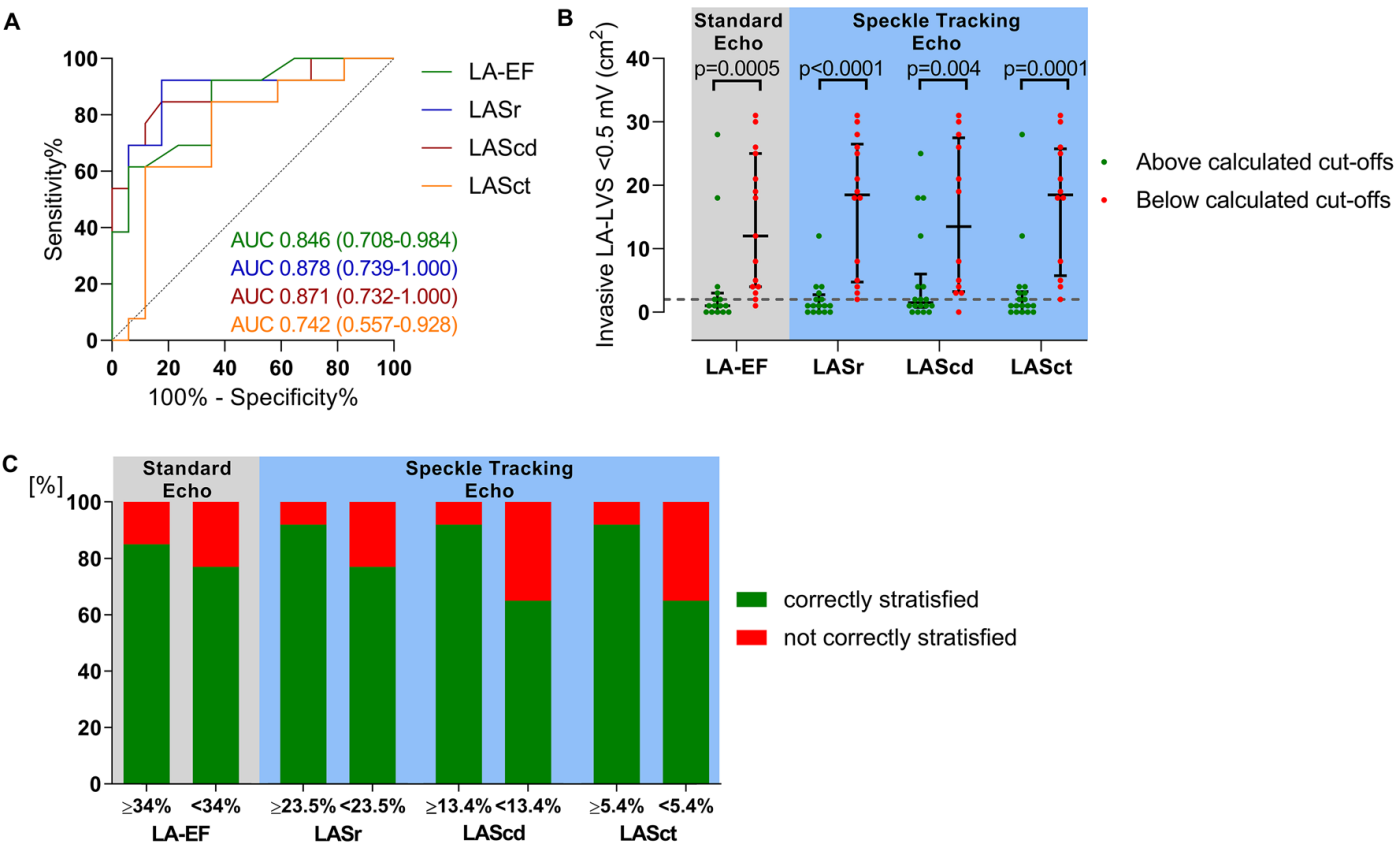
Diagnosis of relevant atrial cardiomyopathy based on echocardiographic parameters. Receiver-operating curves within the derivation cohort determined a left atrial emptying fraction (LA-EF) cut-off of < 34% as predictor for relevant atrial cardiomyopathy (ACM) diagnosis with a sensitivity of 69.2% and a specificity of 76.5%, a left atrial strain in reservoir phase (LASr) < 23.5% (sensitivity of 92.3% and specificity of 82.4%), in conduit phase (LAScd) < 13.4% (sensitivity of 84.6% and specificity of 82.4%) and in contraction phase (LASct) < 5.4% (sensitivity of 61.5% and specificity of 88.2%) **(a)**. Application of these cut-offs to the validation cohort showed a significantly increased left atrial low-voltage substrate (LA-LVS) extent in patients with pathological echocardiography criteria **(b)** and allowed accurate differentiation between patients with and without relevant ACM **(c)**. Whiskers depict median with 25% and 75% interquartile range. Dashed line marks border between absence (< 2 cm^2^ LA-LVS at < 0.5 mV) and presence of relevant ACM

The derivation cohort showed a moderate correlation between ACM extent quantified by endocardial voltage mapping and LA-EF using standard 2D echocardiography (*r* = 0.49, *p* = 0.006). Similar results were obtained for speckle tracking parameters LASr (*r* = 0.51, *p* = 0.004) and LAScd (*r* = 0.54, *p* = 0.002). Correlation of ACM with LASct was low (*r* = 0.30, *p* = 0.1104). ROC analyses for prediction of ACM yielded a c-statistic of 0.846 for LA-EF with the highest sensitivity and specificity obtained at a LA-EF of < 34%. The corresponding cut-off values were < 23.5% for LASr (*c*-statistic of 0.878), < 13.4% for LAScd (*c*-statistic of 0.871) and < 5.4% for LASct (*c*-statistic of 0.742) (Fig. 2a).

### Validation cohort

Patients belonging to the validation cohort showed similar characteristics to those of the derivation cohort with respect to key clinical variables such as age, sex, body mass index (BMI), CHA_2_DS_2_-VASc-score, antiarrhythmic therapy and electrical cardioversion on admission day and ACM extent (Supplementary Online Table 1). Patients with an LA-EF ≥ 34% had a ACM extent of 0.7 cm^2^, whereas ACM extent of patients with a LA-EF < 34% equaled 11.5 cm^2^ (*p* = 0.0005). The corresponding values were 0.7 cm^2^ versus 18.2 cm^2^ for LASr (*p* < 0.0001), 1.4 cm^2^ versus 13.1 cm^2^ for LAScd (*p* = 0.0040) and 1.0 cm^2^ versus 18.2 cm^2^ for LASct (*p* = 0.0001) (Fig. 2b). Accordingly, the determined cut-off values stratified accurately between patients with and without relevant ACM (Fig. 2c): The correct diagnosis of ACM was established in 76% of patients for LA-EF < 34% (positive predictive value: 87%, negative predictive value: 73%) and in 76% of patients with LASr < 23.5% (positive predictive value: 93%, negative predictive value: 75%) as well as in 65% of patients for LAScd < 13.4% (positive predictive value: 92%, negative predictive value: 67%) and in 65% of patients for LASct < 5.4% (positive predictive value: 92%, negative predictive value: 67%).

### Predictors of ACM

**Fig. 3:**
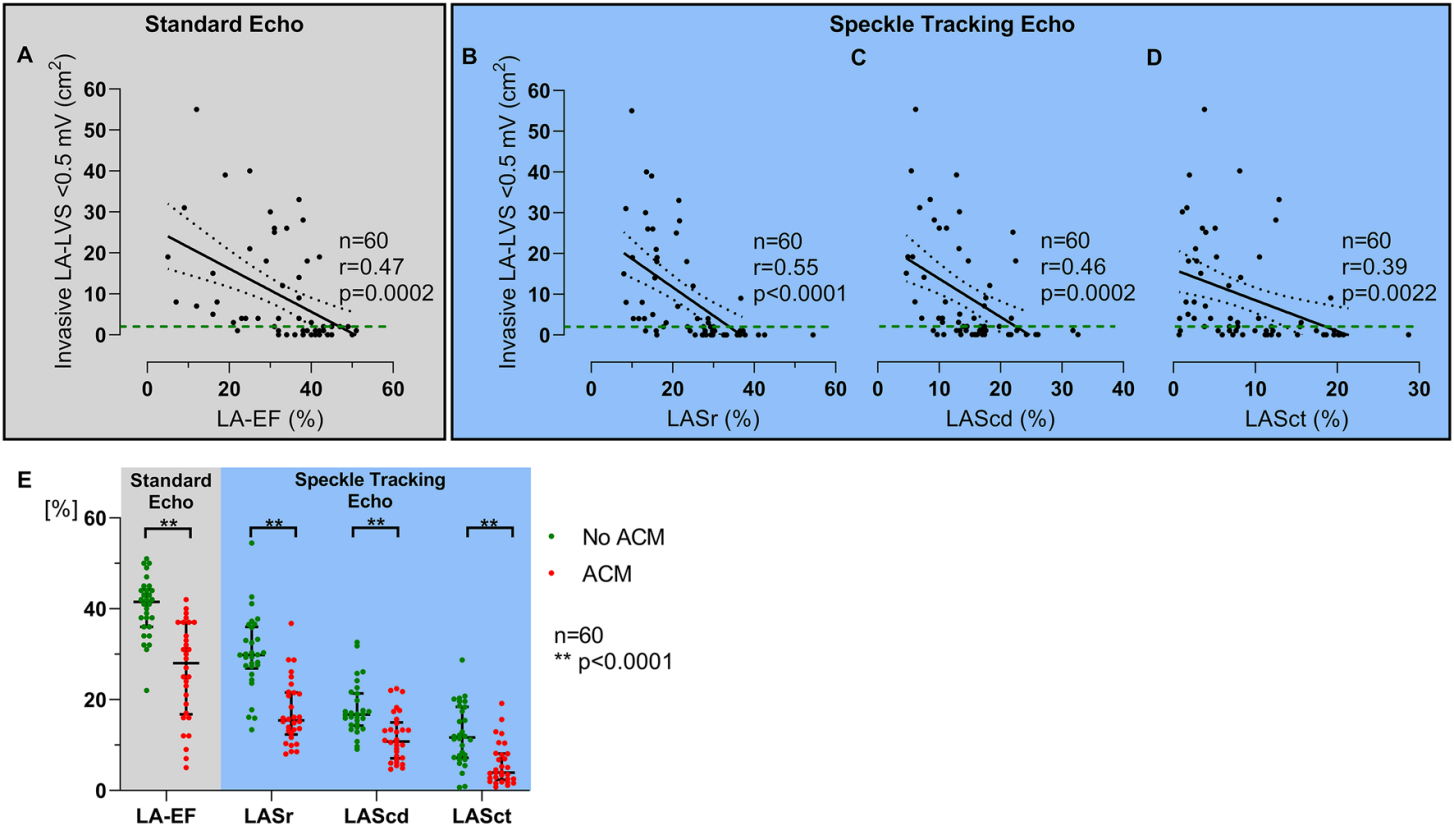
Correlations between echocardiographic parameters and ACM extent. Echocardiographic parameters from both standard left atrial emptying fraction (LA-EF, **a**, left box) and left atrial strain (LAS) parameters (**b–d**, right box) correlated significantly with left atrial low-voltage substrate (LA-LVS). Of all echocardiographic parameters, LAS in reservoir phase (LASr) showed the best correlation with LA-LVS **(b)**. Green dashed line marks border between absence (< 2 cm^2^ LA-LVS extent at < 0.5 mV) and presence of relevant atrial cardiomyopathy (ACM). Linear regression (black line) and 95% confidence bands (black dashed lines) are illustrated. In patients with relevant ACM, LA-EF and LAS parameters were significantly reduced in all phases: 41.5% versus 28.0% for LA-EF, 29.8% versus 15.4% for LASr, 16.7% versus 10.8% for LAScd and 11.7% versus 3.9% for LASct (*p* < 0.0001 for all, **e**)

Univariate analysis for predictors for relevant ACM was based on the whole study cohort. Correlations between ACM extent and echocardiographic parameters are shown in Fig. 3a–d. Patients requiring a second cardioversion on admission (*n* = 14) demonstrate similar correlations to patients remaining in sinus rhythm prior to PVI (Supplementary Online Fig. 2). In patients with ACM, LA-EF and LAS parameters were significantly reduced in all phases (Fig. 3e). Odds ratios for presence of relevant ACM were 15.2 (CI: 4.1–56.2) for LA-EF (*p* < 0.0001), 32.5 (CI: 7.8–135.1) for LASr (*p* < 0.0001), 17.9 (CI: 4.7–67.4) for LAScd (*p* < 0.0001) and 15.6 (CI: 3.8–63.4) for LASct (*p* = 0.0001) in univariate models (Table 2). Furthermore, age, BMI, CHA_2_DS_2_-VASc-Score, LA diameter, LA volume index, and functional mitral regurgitation were associated with relevant ACM in univariate analysis and were included in multivariate models. Adjusted odds ratios were 27.1 (CI: 3.5–212.0) for LA-EF (*p* = 0.002), 27.4 (CI: 3.3–231.4) for LASr (*p* = 0.002), 10.4 (CI: 1.7–62.1) for LAScd (*p* = 0.010) and 12.7 (CI: 2.0–81.3) for LASct (*p* = 0.007).

**Table 2:** Predictors for relevant atrial cardiomyopathy

	Univariate regression analysis		Multivariate regression analysis	
	Odds ratio (95% CI)	*p* value	Odds ratio (95% CI)	*p* value
LA-EF < 34%	15.17 (4.09–56.25)	< 0.001	27.15 (3.48–212.01)	0.002
LASr < 23.5%	32.50 (7.82–135.10)	< 0.001	27.43 (3.25–231.42)	0.002
LAScd < 13.4%	17.88 (4.74–67.43)	< 0.001	10.41 (1.75–62.08)	0.010
LASct < 5.4%	15.55 (3.81–63.36)	< 0.001	12.65 (1.97–81.29)	0.007
Age, years	1.14 (1.05–1.23)	0.001		ns
BMI, kg/m^2^	0.83 (0.71–0.97)	0.016		ns
CHA_2_DS_2_-VASc-Score	1.79 (1.19–2.69)	0.005		ns
LA diameter, mm	1.12 (1.01–1.23)	0.026		ns
LA volume index, mL/m^2^	1.05 (1.00–1.09)	0.037		ns
Functional mitral regurgitation, *n* (%)	8.50 (2.37–30.47)	0.001		ns

*BMI* body mass index, *cd* conduit phase, *ct* contraction phase, *EF* emptying fraction, *LA* left atrial, *LAS* left atrial strain, *r* reservoir phase

*ns* not significant

### Arrhythmia recurrence

**Fig. 4:**
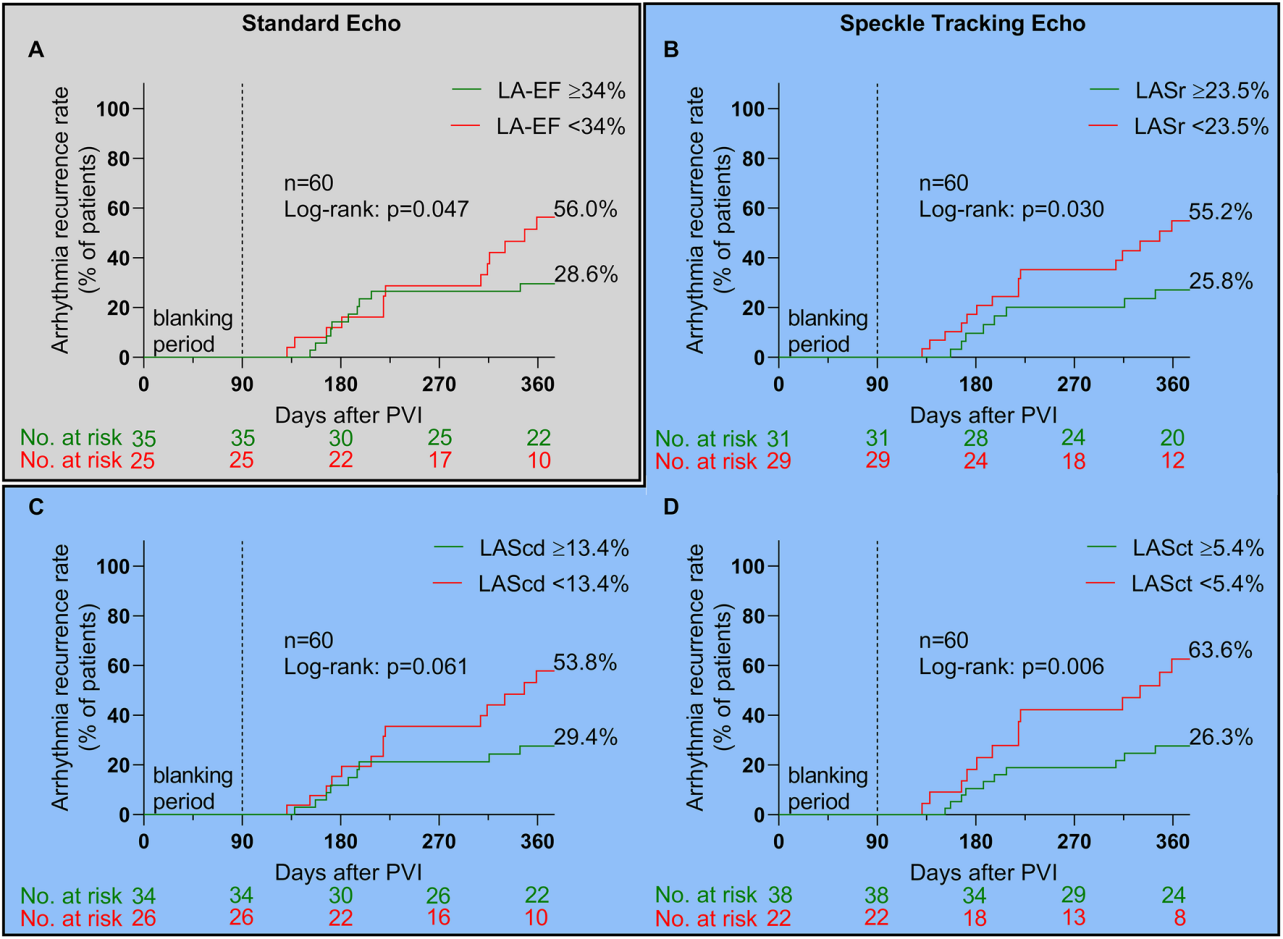
Arrhythmia recurrence after pulmonary vein isolation. Kaplan–Meier curves for arrhythmia recurrences in patients with values above the calculated cut-off values for relevant atrial cardiomyopathy (ACM) (green curves) compared to patients with values below the cut-off values (red curves) are shown in **a–d**. With exception of left atrial strain in conduit phase (LAScd), all echocardiographic cut-offs were able to predict ablation outcome

Arrhythmia recurrence occurred in 24 (40%) patients after a median of 203 (168–316) days during a median follow-up of 355 (200–386) days. Impaired LA-EF, LASr, and LASct were associated with higher arrhythmia recurrence rates within 12 months (log-rank: *p* = 0.047 for LA-EF, *p* = 0.030 for LASr and 0.006 for LASct, respectively, Fig. 4). There was a non-significant trend for higher recurrences with reduced LAScd (log-rank: *p* = 0.061, Fig. 4c). In line with these findings, patients with relevant ACM as diagnosed using endocardial voltage mapping also demonstrated higher arrhythmia recurrence (log-rank: *p* = 0.001; Supplementary Online Fig. 1).

## Discussion

We report three main findings: First, LA function as quantified using standard and speckle tracking echocardiography in sinus rhythm is related to the extent of ACM in patients with AF. Second, echocardiography can stratify patients into those with and those without relevant ACM. Third, echocardiographic ACM diagnosis is of clinical relevance as it predicts arrhythmia recurrence following PVI.

### Atrial cardiomyopathy

Histology of the left atrium can be considered the gold standard for diagnosis of ACM, but is, for obvious reasons, of little clinical relevance [3]. In contrast, endocardial contact mapping is widely available and well established in the context of AF ablation. However, its invasiveness makes it unsuitable for screening and pre-procedural risk stratification [7, 8, 11]. These limitations gave rise to approaches to non-invasively diagnose and quantify ACM: Late gadolinium-enhanced MRI has been evaluated for ACM diagnosis over the last years [6, 9, 10], but standardized acquisition and analysis methods are lacking, which affects reproducibility and the need for extensive expertise. Furthermore, costs are high, the examination is time-consuming and several patient groups have contraindications to MRI (e.g., renal insufficiency, claustrophobia, implanted cardiac devices) [30, 31]. Another recently introduced non-invasive method is measurement of the amplified p-wave duration in sinus rhythm [28, 29]. Its accurate measurement necessitates high-quality (low noise) digital acquisition of 12-lead ECG and high expertise in recognition of the low-voltage components of the amplified p-wave, and it is also limited to patients with sufficient signal-to-noise ratio in ECG.

### Echocardiography and LA function in ACM

Echocardiography has significant potential advantages for ACM diagnosis: It is widely available, relatively easily applicable, and delivers instantaneous results at low cost. Previous studies showed some value for standard and speckle tracking imaging to diagnose ACM: Cameli et al. reported lower LASr in patients with evidence of ACM diagnosed by atrial histology who were undergoing mitral valve surgery for severe regurgitation [18]. The generalizability of these findings, however, may be limited due to the very specific studied patient population. Kuppahally et al., on the other hand, used MRI to detect ACM, and also reported lower LASr in ACM patients [19]. Contrary to the current recommendations of the European Association of Cardiovascular Imaging (EACVI)/American Society of Echocardiography (ASE)/Industry Task Force [27], they limited LASr measurements to specific parts of the LA (the midlateral and midseptal wall) and performed LASr quantifications partly in AF, and partly in sinus rhythm. In the current study, we investigated the usefulness of echocardiography for ACM diagnosis in AF patients. Therefore, we compared echocardiographic LA function to endocardial voltage mapping of the left atrium in sinus rhythm. Our study differs from previous studies in significant ways: First, we used multi-polar, small-electrode mapping catheters, resulting in high-density LA voltage maps with substantially improved delineation of diseased and healthy  atrial tissue as compared to standard low-density mapping with large, single-tip electrode. In contrast to previous studies, this allows a very detailed quantification of the true extent of ACM [20–22]. Second, we performed echocardiography in sinus rhythm only, while previous studies reported strain values which were acquired partly during AF and partly during sinus rhythm [21, 22]. This is of particular importance as the presence of ACM not only affects LA function and, hence, echocardiographic measurements, but also the likelihood that such patients will be in AF during echocardiography. The correlation of functional echocardiographic parameters with ACM may, therefore, be significant due to collinearity of ACM and presence of AF. Third, all patients included in the current study are ablation-naive and present ACM that is associated with the natural course of AF. This is again in contrast to previous reports which included a substantial part of patients who had undergone prior LA ablations causing iatrogenic ACM, which make their findings unsuitable in the context of naturally occurring ACM [32]. Fourth, to the best of our knowledge, this is the first clinical study that defines valid echocardiographic cut-off values for ACM diagnosis using automatic LA strain measurements. A meta-analysis by Pathan et al. reported on values of 39% for LASr, 23% for LAScd and 17% for LASct as being normal in a population including 2,542 healthy subjects from 40 studies [33]. In the current study, we found values of 30% for LASr, 17% for LAScd and 12% for LASct in “healthy” patients without relevant ACM. However, all patients included in our study suffered from AF, which likely contributes to the overall lower values that we observed even in study patients without ACM, as compared to the healthy general population reported by Pathan et al. In patients with ACM, the LAS values are further reduced, allowing echocardiographic diagnosis of ACM.

The current study demonstrates that ACM is associated with presence of functional mitral regurgitation. This is in line with previous studies, which assessed that functional mitral regurgitation is related with new-onset AF, the presence of ACM and arrhythmia recurrence following PVI [26, 34]. It is, however, controversial whether functional mitral regurgitation is a potential cause or the result of ACM. Furthermore, patients with ACM had more frequent evidence for a left ventricular diastolic dysfunction. Several studies also demonstrated an association between diastolic dysfunction and new-onset AF, LA remodeling and AF recurrence following PVI [35–37].

### Clinical relevance of ACM diagnosis

The most recent guidelines emphasize the importance of ACM in the context of new-onset AF and arrhythmia recurrence following AF ablation [2]. The current study underlines these recommendations and validates the use of standard two-dimensional and speckle tracking echocardiography for ACM diagnosis and prediction of arrhythmia recurrence following PVI for persistent AF.

ACM as a disease is not only related to the development and persistency of AF but also seems to play an important role in many cardiovascular pathologies such as heart failure with preserved ejection fraction (HFpEF) [29] and ischemic stroke [6]. Previous studies investigating LAS demonstrated not only an association between reduced LAS values and new-onset AF [12–14] and higher arrhythmia recurrence rates after PVI [15–17], but also an increased risk for HFpEF [38] and stroke [39]. These findings may also be explained by presence of ACM in the investigated patients, resulting in reduced LAS values, and may, therefore, extend the pathophysiological understanding of the above-mentioned cardiovascular diseases.

### Limitations

First, the study’s sample was relatively small. Future large-scale studies are needed to confirm the currently identified cut-off values for LA-EF and LAS for ACM prediction. Second, high quality of the two-dimensional echocardiographic images required for proper quantification of the above-mentioned parameters may be insufficient in some patients. However, no patient was excluded due to insufficient image quality. Third, it is unclear which results manual LA function measurements would have produced compared to those obtained by using automatic strain analysis software as in the current study. Fourth, the cut-off values presented here are valid in sinus rhythm only, requiring cardioversion in certain patients. Sinus rhythm is recommended to ensure automatic analysis due to regular and comparable RR cycles.

## Conclusion

Standard echocardiography and speckle tracking imaging enable accurate diagnosis of ACM and prediction of arrhythmia recurrence after PVI. Therefore, non-invasive echocardiographic measurement of ACM has the potential to become a widespread screening tool in patients with suspected ACM.

## Supplementary Information

Below is the link to the electronic supplementary material.

Supplementary file1 (DOCX 2371 kb)
