# Supplementary material for: Echocardiographic diagnosis of atrial cardiomyopathy allows outcome prediction following pulmonary vein isolation
Source: Clin Res Cardiol. 2021 Apr 29;110(11):1770–80. doi: 10.1007/s00392-021-01850-x (PMC8563528; doi:10.1007/s00392-021-01850-x)
Supplement: Supplementary file 1 — Supplementary file1 (DOCX 2371 kb) [file 392_2021_1850_MOESM1_ESM.docx]

**Supplementary Information:**

Supplementary Table 1: Comparison of clinical and procedural characteristics between the derivation and the validation cohort.

|  | **All patients**  **(n=60)** | **Derivation cohort**  **(n=30)** | **Validation cohort**  **(n=30)** | ***P* value** |
| --- | --- | --- | --- | --- |
| Age, years | 66±9 | 65±10 | 67±8 | 0.36 |
| Male sex, n (%) | 48 (80) | 24 (80) | 24 (80) | 1.0 |
| BMI, kg/m^2^ | 28±4 | 28±4 | 28±4 | 0.74 |
| NYHA functional classification | 2 (2-3) | 2 (2-3) | 2 (2-2) | 0.11 |
| CCS classification | 1 (1-1) | 1 (1-1) | 1 (1-1) | 0.98 |
| Hypertension, n (%) | 41 (68) | 19 (63) | 22 (73) | 0.58 |
| Diabetes mellitus, n (%) | 5 (8) | 1 (3) | 4 (13) | 0.35 |
| Prior stroke or TIA, n (%) | 1 (2) | 1 (3) | 0 (0) | 1.0 |
| Structural cardiomyopathy, n (%) | 10 (17) | 4 (13) | 6 (20) | 0.73 |
| Coronary artery disease, n (%) | 9 (15) | 1 (3) | 8 (27) | 0.03 |
| CHA_2_DS_2_-VASc-Score | 2 (1-3) | 2 (1-3) | 3 (1-3) | 0.28 |
| Prior antiarrhythmic therapy, n (%) | 49 (82) | 21 (70) | 28 (93) | 0.04 |
| Antiarrhythmic therapy on admission day, n (%)  - Amiodarone, n (%)  - Flecainide, n (%)  - Sotalol, n (%)  - Dronedarone, n (%)  - Propafenone, n (%) | 39 (65)  24 (40)  6 (10)  6 (10)  2 (3)  1 (2) | 20 (67)  14 (47)  3 (10)  3 (10)  0 (0)  0 (0) | 19 (63)  10 (33)  3 (10)  3 (10)  2 (7)  1 (3) | 1.0  0.43  1.0  1.0  0.49  1.0 |
| Electrical cardioversion on admission day, n (%) | 14 (23) | 6 (20) | 8 (27) | 0.76 |
| LA diameter, mm | 46±6 | 46±6 | 45±7 | 0.73 |
| LA volume index, mL/m^2^ | 49±14 | 51±15 | 46±12 | 0.14 |
| LA-EF, % | 37 (26-42) | 37 (24-42) | 35 (29-41) | 0.89 |
| LASr  - 4 C  - 2C  - averaged | 24 (16-33)  22 (15-32)  24 (15-30) | 23 (14-32)  22 (13-35)  24 (15-33) | 24 (16-33)  22 (16-29)  24 (16-29) | 0.76  0.82  0.89 |
| LAScd  - 4 C  - 2C  - averaged | 15 (10-18)  14 (9-18)  14 (10-18) | 14 (9-18)  13 (8-17)  13 (9-17) | 15 (11-18)  15 (10-18)  15 (11-19) | 0.45  0.22  0.32 |
| LASct  - 4 C  - 2C  - averaged | 7 (4-13)  8 (3-14)  8 (4-13) | 7 (3-13)  9 (4-16)  8 (4-15) | 8 (4-12)  7 (3-13)  7 (3-12) | 0.78  0.29  0.48 |
| LVEF, % | 57±8 | 57±7 | 57±8 | 0.84 |
| LV dysfunction with LVEF <50%, n (%) | 12 (20) | 5 (17) | 7 (23) | 0.75 |
| LVEDD, mm | 54±5 | 53±5 | 54±6 | 0.69 |
| LV cavity dilatation, n (%) | 12 (20) | 5 (17) | 7 (23) | 0.75 |
| LV strain, % | 18±3 | 18±3 | 18±3 | 0.86 |
| PAP, mmHg * | 33 (27-38) | 35 (28-41) | 32 (26-35) | 0.34 |
| E/A | 1.48 (1.03-2.25) | 1.41 (1.07-2.16) | 1.60 (0.94-2.41) | 0.71 |
| DT, s | 0.205 (0.180-0.240) | 0.205 (0.183-0.246) | 0.205 (0.171-0.240) | 0.62 |
| Septal e’, cm/s | 7.00 (5.00-8.25) | 6.25 (5.00-8.00) | 7.15 (5.75-9.00) | 0.21 |
| E/e’ | 10.89 (8.37-14.00) | 11.83 (8.71-14.75) | 10.47 (8.17-13.55) | 0.41 |
| Relevant (at least moderate) mitral valve regurgitation, n (%) | 4 (7) | 3 (10) | 1 (3) | 0.61 |
| Functional mitral regurgitation, n (%) | 21 (35) | 14 (47) | 7 (23) | 0.10 |
| LA-LVS at <0.5 mV, cm^2^ | 2.5 (0.4-17.0) | 1.4 (0.2-14.5) | 3.7 (0.7-18.0) | 0.34 |

BMI: body mass index, C: chamber, cd: conduit phase, ct: contraction phase, DT: deceleration time, EDD: end-diastolic diameter, LA: left atrial, LA-EF: left atrial emptying fraction, LVEF: left ventricular ejection fraction, LAS: left atrial strain, LV left ventricular, LVS: low-voltage-substrate, PAP: pulmonary artery pressure, r: reservoir phase, TIA: transient ischemic attack

* measurable in 42/60 patients (70%)

**Supplementary Fig. 1** Arrhythmia recurrence after pulmonary vein isolation in patients with relevant ACM


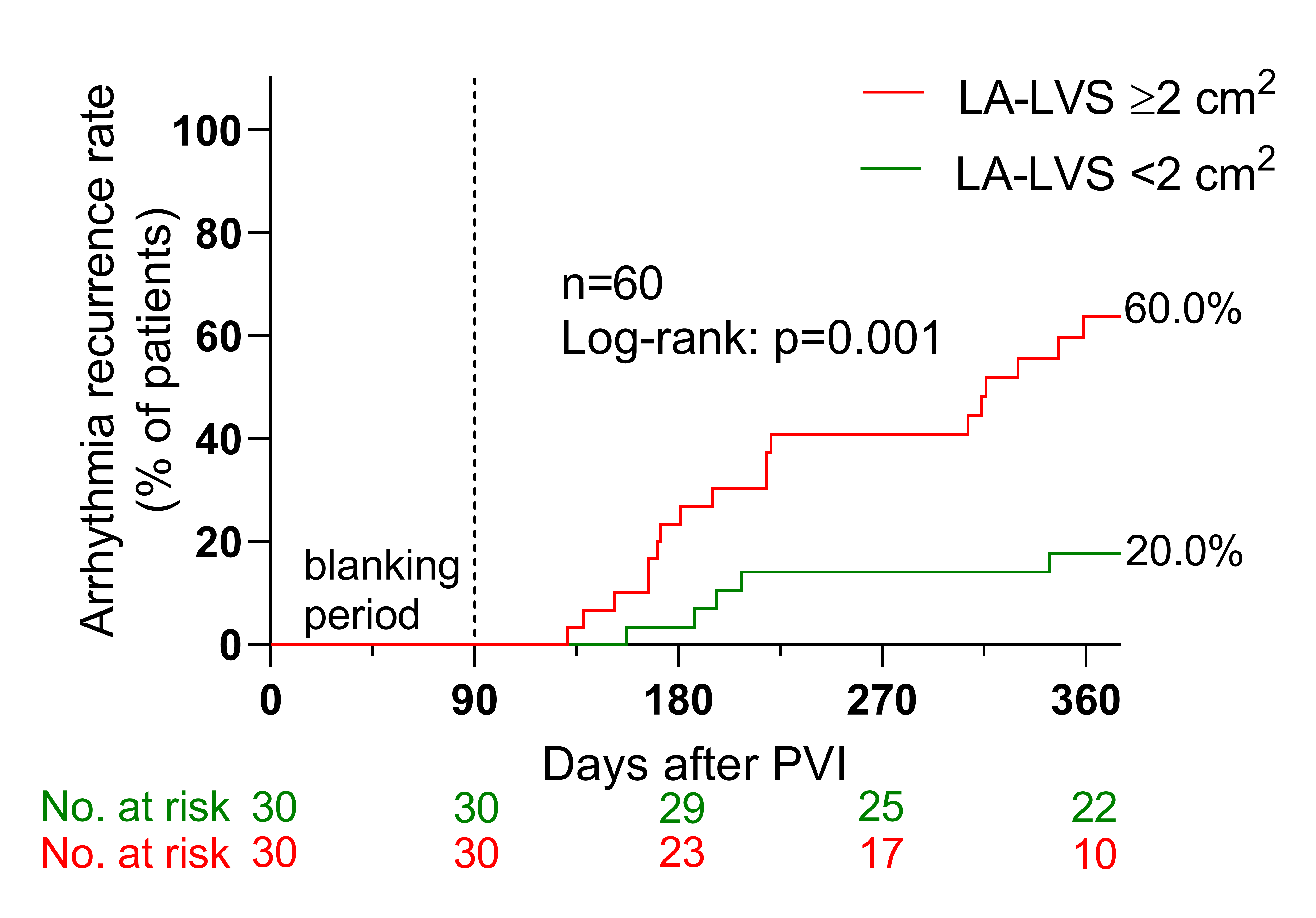


Kaplan-Meier curves for arrhythmia recurrences in patients without relevant atrial cardiomyopathy (ACM, left atrial low-voltage substrate (LA-LVS) <2 cm^2^, green curve) compared to patients with relevant ACM (LA-LVS ≥2 cm^2^, red curve) are shown.

**Supplementary Fig. 2** Impact of electrical cardioversion at admission on left atrial function





Impact of electrical cardioversion (DC) at admission (n=14 patients) on correlation between echocardiographic parameters and left atrial low-voltage substrate (LA-LVS) is depicted. Green dashed line marks border between absence (<2cm^2^ LA-LVS extent at <0.5mV) and presence of relevant atrial cardiomyopathy (ACM). Linear regression (black line) and 95% confidence bands (black dashed lines) are illustrated. No statistically significant difference could be observed for both left atrial emptying fraction (LA-EF, Panel A) and left atrial strain parameters (LAS, Panels B-D) in patients with electrical cardioversion on admission day (small squares) versus patients with prior electrical cardioversion (small circles).
